# Dog owners are more likely to meet physical activity guidelines than people without a dog: An investigation of the association between dog ownership and physical activity levels in a UK community

**DOI:** 10.1038/s41598-019-41254-6

**Published:** 2019-04-18

**Authors:** Carri Westgarth, Robert M. Christley, Christopher Jewell, Alexander J. German, Lynne M. Boddy, Hayley E. Christian

**Affiliations:** 1 Institute of Infection and Global Health, University of Liverpool, Liverpool, UK; 2 Institute of Veterinary Science, University of Liverpool, Liverpool, UK; 3 Lancaster Medical School, Lancaster University, Lancaster, UK; 4 Institute of Ageing and Chronic Disease, University of Liverpool, Liverpool, UK; 5 School of Sport and Exercise Science, Liverpool John Moores University, Liverpool, UK; 6 School of Population and Global Health, The University of Western Australia, Perth, Australia

**Keywords:** Health policy, Epidemiology, Weight management

## Abstract

Previous research suggests that dog owners are slightly more physically active than those without dogs, but have only studied one household member, and it is unclear whether time spent dog walking replaces other physical activity (PA). A survey of 191 dog owning adults (DO), 455 non-dog owning adults (NDO), and 46 children, living in 385 households in West Cheshire UK, was conducted in July-August 2015. Objective (accelerometer) validation occurred on a subset (n = 28 adults). Survey PA outcomes were modelled using hierarchical logistic and linear multivariable regression modelling, accounting for clustering of participants in households. DO were far more likely than NDO to report walking for recreation (OR = 14.35, 95% CI = 5.77–35.79, P < 0.001), and amongst recreational walkers walked for longer per week (RR = 1.39, 95% CI = 1.27–5.91, P < 0.001). Other PA undertaken did not differ by dog ownership. The odds of DO meeting current physical activity guidelines of 150 mins per week were four times greater than for NDO (OR = 4.10, 95% CI = 2.05–8.19, P < 0.001). Children with dogs reported more minutes of walking (P = 0.01) and free-time (unstructured) activity (P < 0.01). Dog ownership is associated with more recreational walking and considerably greater odds of meeting PA guidelines. Policies regarding public spaces and housing should support dog ownership due to PA benefits.

## Introduction

Dog ownership is of public health interest due to the potential to promote health-enhancing physical activity (PA) and improved cardiovascular outcomes^1^. Evidence suggests dog ownership is associated with lower risk of death, and a lower risk of cardiovascular conditions at least in single-person households, where the participant may be more highly obligated to dog walk^2^. It is recommended that adults undertake at least 150 minutes of moderate-to-vigorous intensity physical activity (MVPA) per week^3^, but this is achieved by only 66% of men and 58% of women in the England^4^, and under 50% of US adults^5^. A 2013 review concluded considerable evidence that dog owners were more physically active than people without a dog with small to moderate effect sizes^1^. However findings from some studies have been inconsistent, mainly because some owners do not walk with their dogs^6,7^. Nevertheless, considering the number of households that own dogs (e.g 24% UK^8^, 48% USA^9^, and 39% Australia^10^), even small effect sizes might contribute considerable additional physical activity at the population level provided, of course, that the dogs are actually walked.

The different types of exercise that dog owners (DO) and non-dog owners (NDO) report participating in requires investigation. Dog walking is reported to be the only physical activity for some owners but for others it limits other activity (potentially of higher intensity) as ‘there are only so many hours in the day’ and the dog takes priority^11^. However, there is some evidence that participation in other types of MVPA is also greater in DO than NDO^12^. It is also not known what proportion of dog walking is undertaken for recreational reasons, and what proportion is dispersed practice^13^ e.g. primarily transport-related activity, such as walking to a local shop, to drop/pick up from school, or to a work place. Anecdotally, other physical activities with a dog are also popular, such as jogging or cycling, but it is not known how common these are.

It is difficult to compare dog walking rates directly between countries as study designs and measures vary, but UK owners potentially participate in more dog walking than North America and Australia where most previous research has been conducted^1^, due to social and climatic differences. Daily walking of dogs is the accepted social expectation in the UK^11^ with this occurring for 78% of dogs in a UK study^14^. A common reason reported by USA dog owners for not walking their dog was that the dog self-exercised or was an outside dog (43%)^7^, and warm climates in Australia may have a similar effect. In contrast, only 4% of pet dogs in a UK community slept outside^15^. Dog owners are also more highly motivated to walk in bad weather than their non-dog owning counterparts^16^, which could be advantageous for dog owners’ activity levels given the often cool and wet weather in the UK. Specific UK research has focused on older adults^16^; pregnant women^17^; children^18^; and adolescents^19^. To the author’s knowledge, no studies have investigated the association between dog ownership and PA in a general adult population, and this study aims to fill this gap. This will allow cross-country comparisons and contribute to the development of robust intervention strategies to promote dog walking across different countries.

Therefore the first aim of this study was to compare the physical activity of dog owners from UK population with people that do not own a dog. This study is superior to those previously conducted on dog walking in a number of ways. It uses both self-report and objective measures of physical activity, as people have a tendency to over-report physical activity on surveys. Research often focuses on one participant per household, potentially biased towards the person with the most involvement in dog care, inflating impact of dogs. In contrast, this study attempted to recruit and assess the PA of all household members, including children. Another unique aspect of this study was that all of the participants resided in the same community and thus had access to the same neighbourhood environment for walking, known to influence activity levels^20,21^, although perhaps only in dog owners^22^. Therefore this is the first study of dog ownership to truly account for perceived differences in PA that may actually be attributed to dog owners living in different environments to people without a dog. A final novel aspect of this study is the methods of analysis used. Parametric linear regression methods are not strictly appropriate for analysis of PA data, despite often being undertaken^23^. Our analysis methods address this issue, providing more accurate estimates of the effects of dog ownership. We hypothesised that dog owners (DO) would be more likely to meet PA guidelines than non-dog owners (NDO), and the effect sizes would be greater than reported previously (which were odds ratios (OR) less than 2)^12,24^. A secondary aim of the study was to investigate whether DO spend more or less time than NDO in more intensive PA than walking. We hypothesised that increased PA in dog owners would be additional to, and not replacing other forms of activity.

## Results

Responses were received from 385 (55.2%) households with 694 (43.6%) participants (total household response rate 30.1% of study area (1280 households)). Sociodemographic descriptors of the adult participants are given in Table 1. There were slightly more female than males and participants were mainly middle-aged or older adults. Dog owners were significantly younger, more likely to work, had higher household gross income, slightly different education patterns, and had higher self-rated health (all P < 0.05).

**Table 1:** Demographics of survey sample presented as adult non-dog owners (NDO, n = 455) and dog owners (DO, n = 191), residing in 385 households in West Cheshire, UK, 2015. DO can be further split into dog-walkers (DW, n = 169) and non-dog walkers (NDW, n = 18).

Variable		NDO	DO	NDW	DW	P NDO/DO	P NDO/NDW/DW
		% (n)	% (n)	% (n)	% (n)		
***Household factors***							
House type	Detached	71.3 (316)	69.5 (130)	64.7 (11)	70.5 (117)	0.90	0.40
	Semi-detached	20.3 (90)	21.4 (40)	35.3 (6)	19.3 (32)		
	Terraced	8.4 (37)	9.1 (17)	0 (0)	10.2 (17)		
Number of people in household	1	17.4 (79)	12.0 (23)	5.6 (1)	13.0 (22)	0.16	0.27
	2	51.2 (232)	51.3 (98)	44.4 (8)	51.5 (87)		
	3+	31.4 (142)	36.7 (70)	50.0 (9)	35.5 (60)		
Children present in household (<16)	No	84.9 (392)	89.5 (170)	94.4 (17)	88.7 (149)	0.12	0.28
	Yes	15.1 (68)	10.5 (20)	5.6 (1)	11.3 (19)		
***Personal factors***							
Gender	Male	46.5 (208)	42.9 (81)	33.3 (6)	45.1 (72)	0.40	0.44
	Female	53.5 (239)	57.1 (108)	66.7 (12)	56.9 (95)		
	OR (95% CI)	1	1.16 (0.82–1.63)				
Age categorised	<30	7.2 (32)	13.5 (26)	38.9 (7)	11.4 (19)	**0.01**	**<0.001**
	30–49	21.4 (95)	17.5 (33)	5.6 (1)	19.2 (32)		
	50–69	44.8 (199)	32.1 (94)	38.9 (7)	51.5 (86)		
	70+	26.6 (118)	23.4 (36)	16.7 (3)	18.0 (30)		
Marital status	Not	29.4 (131)	24.2 (46)	44.4 (8)	22.6 (38)	0.18	0.07
	Married or living with partner	70.6 (315)	75.8 (144)	55.6 (10)	77.4 (130)		
***Socio-economic factors***							
Highest Education	Other school leaving certificate or none	21.7 (95)	14.1 (26)	6.3 (1)	14.5 (24)	**0.004**	**0.03**
	GCSE or O’level equivalent (level of High-School Diploma)	20.6 (90)	31.9 (59)	37.5 (6)	30.7 (51)		
	A-level or equivalent (level of US Advanced Placement)	10.3 (45)	13.5 (25)	18.8 (3)	13.3 (22)		
	Degree/diploma or above	47.5 (208)	40.5 (75)	37.5 (6)	41.6 (69)		
Work status	None/home/retired	53.6 (238)	40.9 (77)	29.4 (5)	40.7 (68)	**0.004**	**0.004**
	Working or studying (Full or part-time, paid or unpaid)	46.4 (206)	59.0 (111)	70.6 (12)	59.3 (99)		
Household gross income	£0–20,000 ($0–27,000)	29.3 (110)	21.4 (31)	11.1 (1)	21.8 (29)	**0.03**	—
	£20–40,000 ($27–54,000)	36.3 (136)	30.3 (44)	22.2 (2)	30.1 (40)		
	£40–60,000 ($54–81,000)	20.3 (76)	30.3 (44)	44.4 (4)	30.1 (40)		
	£60,000+ ($81,000+)	14.3 (53)	17.9 (26)	22.2 (4)	18.1 (24)		
***Health factors***							
Physically active at work	No	43.7 (90)	43.5 (47)	45.5 (5)	43.3 (42)	0.98	0.99
	Yes	56.3 (116)	56.5 (61)	54.5 (6)	56.7 (55)		
Physically active at work/work status combined	Physically inactive at work	20.5 (91)	26.6 (50)	35.3 (6)	26.4 (44)	**0.01**	**0.03**
	Physically active at work	25.9 (115)	32.5 (61)	35.3 (6)	32.9 (55)		
	Does not work	53.6 (238)	41.0 (77)	29.4 (5)	40.7 (68)		
Self-rated general health	Poor-good	61.2 (273)	45.5 (86)	27.8 (5)	46.7 (78)	**0.000**	**0.000**
	Very good-excellent	38.8 (173)	54.5 (103)	72.2 (13)	53.3 (89)		
	OR (95% CI)	1	1.89 (1.34–2.67)				
Weight status	Normal or below	45.5 (191)	43.7 (76)	61.5 (8)	43.3 (68)	0.91	0.51
	Overweight	37.4 (157)	39.1 (68)	38.5 (5)	38.3 (60)		
	Obese	17.1 (72)	17.2 (30)	0 (0)	18.5 (29)		
***Other factors***		**Median (n)**	**Median (n)**				
Self-rated personality (TIPI 1-7)	Extraversion	4 (419)	4.5 (180)	3.5 (14)	4/5 (163)	0.17	0.11
	Agreeableness	5.5 (414)	5.5 (176)	5.8 (12)	5.5 (161)	0.78	0.77
	Conscientiousness	6.0 (415)	5.8 (178)	5.0 (14)	6.0 (161)	0.09	0.18
	Emotional Stability	5.0 (414)	5.0 (180)	4.0 (14)	5.0 (163)	0.39	0.08
	Open to Experiences	5.0 (414)	5.0 (177)	4.5 (13)	5.0 (161)	0.45	0.42
Family social support for walking	Low-high	2 (414)	2 (185)	1 (16)	2 (165)	**0.01**	**0.004**
Friend social support for walking	Low-high	0 (425)	0 (181)	0 (15)	0 (162)	0.20	0.41

OR = Odds Ratio. TIPI = Ten-Item Personality Inventory.

### Dog-related physical activity in adults

Dog owners walked with their dogs a median 7.0 times per week (range 0–32) and for a median 220.0 mins per week (range 0–1755). However, eighteen people (9.6%) who owned a dog reported 0 mins walking with their dog; excluding these non-dog walkers (NDW) increased the median time spent dog walking for dog walkers (DW) to 248 mins per week (range 10–1755). Dog walking was mostly done for recreation, health and fitness (median 210 mins per week, (range 0–1680) compared with 0 mins (range 0–840) for transport); 33 dog owners (17.6%) reported walking their dogs for transport, 10 (5.3%) jogging with their dogs and 4 (2.1%) cycling with their dogs. Overall, dog owners spent a median 248 mins per week (range 0–3100) participating in PA with their dog. Sixty-four percent of dog owners met the PA guidelines through their dog walking alone (71% of dog walkers).

### Descriptive analysis unadjusted

Comparisons for self-reported PA outcomes in adults are presented in Table 2 ((NDO and DO) and (NDO, NDW and DW)). It is worth noting that NDW had very low levels of PA; only 29% of NDW met PA guidelines compared with over 80% of all DO, (88% of DW) and 62% of NDO (P < 0.001). Walking for recreation contributed a median of 67% of the total PA for dog owners compared to only 31% for those without a dog (P < 0.001). Dog owners were more likely to report jogging/running without a dog (P = 0.03) and less likely to report Yoga/Pilates (P = 0.03); see Table 3. No other differences in PA types were found.

**Table 2:** Self reported physical activity outcomes adults raw unadjusted for NDO (Non-Dog Owners) vs DO (Dog Owners), and NDO vs NDW (Non-Dog Walkers) vs DW (Dog Walkers), residing in 385 households in West Cheshire, UK, 2015.

Outcome	NDO			DO			P Med NDO/DO	P Mean NDO/DO	NDW			DW			P Med NDO/NDW/DW	P Mean NDO/NDW/DW
	n	Med (IQR)	Mean(SD)	n	Med (IQR)	Mean (SD)			n	Med (IQR)	Mean (SD)	n	Med (IQR)	Mean (SD)		
Walk for recreation frequency/week	449	1 (2)	1.6 (2.2)	187	7 (8)	7.3 (6.0)	**0.000**	**0.000**	18	0 (0)	0.7 (1.9)	169	7 (9)	7.9 (5.6)	**0.000**	**0.000**
Walk for recreation mins/week	445	30 (120)	84 (136)	184	210 (360)	293 (300)	**0.000**	**0.000**	18	0 (0)	27.8 (65.5)	166	240 (325)	322.3 (301.7)	**0.000**	**0.000**
Walk for transport frequency/week	449	2 (5)	3.0 (3.7)	187	0 (3)	2.4 (4.5)	**0.000**	0.14	18	0 (1.3)	1.3 (3.3)	169	0 (3)	2.5 (4.6)	**0.000**	0.13
Walk for transport mins/week	444	40 (90)	75 (123)	186	0 (60)	53 (113)	**0.000**	**0.000**	18	0 (11.3)	15.8 (42.6)	168	0 (60)	56.8 (117.7)	**0.000**	**0.04**
Total walk frequency/week	449	4 (5)	4.6 (4.6)	187	7 (10)	9.6 (8.0)	**0.000**	**0.000**	18	0 (2.5)	2.1 (3.7)	169	8 (9)	10.4 (7.9)	**0.000**	**0.000**
Total walk mins/week	442	90 (190)	159 (209)	184	250 (372.5)	347 (316)	**0.000**	**0.000**	18	0 (60)	43.6 (73.5)	166	292.5 (355)	379.7 (315.1)	**0.000**	**0.000**
MVPA freq/week	449	1 (4)	2.2 (2.9)	187	2 (4)	2.9 (5.1)	0.17	0.09	18	0.5 (4)	2.0 (2.6)	169	2 (5)	3.0 (5.3)	0.23	0.06
MVPA mins/week	441	60 (180)	127 (190)	179	60 (200)	126 (180)	0.97	0.97	17	0 (120)	80.2 (124.9)	162	60 (200)	131.4 (184.3)	0.44	0.56
VPA freq/week	449	0 (1)	0.7 (1.5)	187	0 (1)	0.9 (1.7)	0.50	0.16	18	0 (2)	0.9 (1.6)	169	0 (1)	0.9 (1.7)	0.76	0.32
VPA mins/week	448	0 (30)	37.1 (91.4)	183	0 (30)	51 (119)	0.78	0.15	18	0 (52.5)	52.2 (103.0)	165	0 (40)	51.0 (120.5)	0.88	0.28
Total PA mins/week	439	205 (340)	286 (293)	176	420 (440)	476 (357)	**0.000**	**0.000**	17	75 (210)	126.4 (156.6)	159	440 (480)	515.3 (352.3)	**0.000**	**0.000**
% of total PA walking contributes	397	61.9 (68.9)	59.8 (35.8)	171	83.3 (40.9)	73.4 (30.5)	**0.000**	**0.000**	12	35.3 (100)	45.9 (43.9)	159	84.2 (39.0)	75.5 (28.3)	**0.000**	**0.000**
% of total PA walking for recreation contributes	397	20.8 (45.5)	27.8 (29.9)	171	66.7 (60.9)	60.0 (33.7)	**0.000**	**0.000**	12	0.0 (45.0)	21.3 (39.6)	159	67.1 (56.3)	63.0 (31.4)	**0.000**	**0.000**
% of total PA walking for transport contributes	397	22.2 (50.0)	32.0 (33.3)	171	0.0 (17.7)	13.4 (23.0)	**0.000**	**0.000**	12	2.4 (36.4)	24.6 (37.7)	159	0.0 (17.4)	12.5 (21.5)	**0.000**	**0.000**
% of total walking dog walking contributes					100 (77.9–100.0)	84.5 (26.6)							100 (82.5–100)	88.6 (19.4)		
% of total physical activity dog walking contributes					71.4 (42.9–94.6)	65.0 (32.3)							75.2 (51.2–95.3)	69.1 (28.6)		

DO: dog owners; NDO: non-dog owners; NDW: non-dog walkers; DW: dog walkers; PA: physical activity; MVPA: moderate-vigorous intensity physical activity; VPA vigorous physical activity; Med:median.

**Table 3:** Activity types (other than walking) reported participated in (unadjusted), by participants (dog –Owning (DO) and non-dog owning (NDO), residing in 385 households in West Cheshire, UK, 2015.

Activity		NDO	DO	P NDO/DO	NDO	NDW	DW	P NDO/NDW/DW
		% (n)	% (n)		% (n)	% (n)	% (n)	
Jog/run	No	95.1 (431)	90.5 (171)	**0.03**	95.1 (431)	88.9 (16)	90.5 (153)	0.07
	Yes	4.9 (22)	9.5 (18)		4.9 (22)	11.1 (2)	9.5 (16)	
	OR (95% CI)	1	2.06 (1.08–3.94)					
Swimming	No	89.0 (403)	89.4 (169)	0.87	89.0 (403)	100 (18)	88.2 (149)	0.31
	Yes	11.0 (50)	10.6 (20)		11.0 (50)	0 (0)	11.8 (20)	
	OR (95% CI)	1	0.95 (0.55–1.65)					
Cycling	No	79.9 (362)	83.1 (157)	0.35	79.9 (362)	94.4 (17)	81.7 (138)	0.30
	Yes	20.1 (91)	16.9 (32)		20.1 (91)	5.6 (1)	18.3 (31)	
	OR (95% CI)	1	0.81 (0.52–1.26)					
Aerobics/dance	No	89.9 (407)	88.9 (168)	0.72	89.9 (407)	88.9 (16)	88.8 (150)	0.92
	Yes	10.1 (46)	11.1 (21)		10.1 (46)	11.1 (2)	11.2 (19)	
	OR (95% CI)	1	1.11 (0.64–1.91)					
Gym session	No	87.6 (397)	85.7 (162)	0.51	87.6 (397)	88.9 (16)	85.2 (144)	0.70
	Yes	12.4 (56)	14.3 (27)		12.4 (56)	11.1 (2)	17.8 (25)	
	OR (95% CI)	1	1.18 (0.72–1.94)					
Individual sport	No	92.3 (418)	92.6 (175)	0.89	92.3 (418)	94.4 (17)	92.3 (156)	0.94
	Yes	7.7 (35)	7.4 (14)		7.7 (35)	5.6 (1)	3.7 (13)	
	OR (95% CI)	1	0.96 (0.50–1.82)					
Team sport	No	94.9 (430)	95.2 (180)	0.87	94.9 (430)	72.2 (13)	97.6 (165)	—
	Yes	5.1 (23)	4.8 (9)		5.1 (23)	27.8 (5)	2.4 (4)	
	OR (95% CI)	1	0.93 (0.42–2.06)					
Gardening and housework	No	67.8 (307)	70.9 (134)	0.44	67.8 (307)	83.3 (15)	69.8 (118)	0.35
	Yes	32.2 (146)	29.1 (55)		32.2 (146)	16.7 (3)	30.2 (51)	
	OR (95% CI)	1	0.86 (0.60–1.25)					
Horse riding	No	99.1 (449)	97.9 (185)	0.24	99.1 (449)	100 (18)	97.6 (165)	—
	Yes	0.88 (4)	2.1 (4)		0.9 (1)	0 (0)	2.4 (4)	
	OR (95% CI)	1	2.43 (0.60–9.81)					
Yoga/Pilates	No	96.5 (437)	99.5 (188)	**0.03**	96.5 (437)	100 (18)	99.4 (168)	—
	Yes	3.5 (16)	0.5 (1)		3.5 (16)	0 (0)	0.6 (1)	
	OR (95% CI)		0.15 (0.02–1.10)					
Other activity	No	98.5 (446)	98.4 (186)	1.0	98.5 (446)	100 (18)	98.2 (166)	—
	Yes	1.6 (7)	1.6 (3)		1.5 (7)	0 (0)	1.8 (3)	
	OR (95% CI)		1.03 (0.26–4.02)					

Table 4 presents the unadjusted accelerometry findings for 28 adults (11 NDO and 17 DW). A non-significant but relevant effect size was found; dog walkers measured 2000 more steps and 13 more minutes of moderate-to-vigorous physical activity per day compared to non-owners (P = 0.34 and 0.37 respectively). Of the six dog owners who reported walking with their dogs some days but not others, a mean 3010 extra steps per day (range 691–7236) were reported on dog walking days.

**Table 4:** Accelerometry physical activity objective measures of 28 participants, in West Cheshire UK, 2015.

	NDO*	Mean (SD)	DW*	Mean (SD)	Difference medians	Difference means	P Medians NDO/DW	P Means NDO/DW
	Median (IQR)		Median (IQR)					
n	11		17					
Average steps/day	6036 (4606)	6381 (3215)	8038 (33663)	7523 (2710)	2002	1142	0.41	0.34
Average CPM Axis 1	321.6 (174.9)	286.2 (111.6)	375.4 (132.8)	339.2 (101.1)	53.8	53.0	0.20	0.22
Average % Sedentary	67.5 (15.6)	66.8 (9.3)	65.8 (10.5)	64.11 (9.3)	−1.7	−2.7	0.41	0.47
Average %LMVPA	32.5 (15.6)	33.3 (9.3)	34.2 (10.5)	35.9 (9.3)	1.7	2.6	0.42	0.47
Average %MVPA	3.1 (3.6)	3.6 (2.4)	4.9 (3.6)	4.5 (2.3)	1.8	0.9	0.27	0.34
Average LMVPA mins/day	287.1 (147.6)	276.1 (97.6)	314.4 (72.0)	297.1 (70.2)	27.3	30.0	0.59	0.54
Average MVPA mins/day	26.6 (21.3)	30.3 (21.4)	39.1 (31.5)	37.8 (20.3)	12.5	7.5	0.23	0.37
Projected average mins MVPA/week	186.0 (149.0)	211.8 (150.1)	274.0 (220.5)	264.4 (141.8)	88	52.6	0.23	0.37
	n	%	n	%			Fisher’s P	
% that would meet PA guidelines (150 mins/week MVPA)	7	63.6	13	76.5			0.67	

DW: *Dog Walker; IQR: interquartile range; NDO: non-dog owner; PA: physical activity; MVPA: moderate-vigorous intensity physical activity; LMVPA: Light-moderate-vigorous physical activity; CPM: counts per minute.

*The category Dog Walker (DW) has been used instead of Dog Owner: One NDO was reclassified as a DW as she was looking after a family member’s dog during the study period and one NDO was reclassified as DW as she regularly walked a neighbour’s dog during the study period.

### Multivariable modelling

The addition of weight status and perceived general health made very little difference to the model estimates so only the findings from model 1 are presented in Table 5. The odds of walking for transport was lower in DO compared with NDO (OR 0.32, 95% CI 0.19–0.53), but if walking for transport occurred, there was no difference in the duration per week between NDO and DO. Dog owners were 14 times more likely than non-owners to walk for recreation (OR 14.35, 95% CI 5.77–35.79) and amongst people who walked for recreation, dog owners also walked for 39% more minutes per week (RR = 1.39, 95% CI 1.27–5.91). In contrast, there was no evidence that participation in other MVPA activities were more or less likely in dog owners, nor of longer or shorter duration per week if they were undertaken. Overall, The odds of DO meeting current physical activity guidelines of 150 mins per week were four times greater than for NDO (OR = 4.10, 95% CI 2.05–8.19). This represents an absolute difference of 87.3% of DO achieving 150 mins per week compared to 62.7% of NDO. In all but two cases the self-report and objective measures provided the same outcome in terms of meeting guidelines. Two participants met guidelines by self-report but not accelerometry, 20 met guidelines by both measures, and 6 did not meet guidelines by accelerometry or self-report.

**Table 5:** Univariable and multivariable hierarchical logistic and linear regression modelling in non-dog owners and dog-owners, of odds of undertaking physical activity and relative risk in minutes if that physical activity type occurs, in a study of participants residing in 385 households in in West Cheshire, UK, 2015.

		Univariable	P	Adjusted	P
		OR/RR (95% CI)		OR/RR (95% CI)	
Walking for transport	OR No/yes	0.56 (0.22–0.58)	<0.001	0.32 (0.19–0.53)	<0.001
	RR Minutes if	0.95 (0.85–1.10)	0.41	0.95 (0.84–1.08)	0.43
Walking for recreation	OR No/yes	18.23 (6.9–48.2)	<0.001	14.35 (5.77–35.79)	<0.001
	RR Minutes if	1.37 (1.25–1.51)	<0.001	1.39 (1.27–5.91	<0.001
Total walking	OR No/yes	9.14 (0.81–102.63)	0.07	8.71 (2.85–26.65)	<0.001
	RR Minutes if	1.31 (1.20–1.44)	<0.001	1.30 (1.19–1.43)	<0.001
MVPA	OR No/yes	1.16 (0.65–2.09)	0.62	1.12 (0.59–2.12)	0.73
	RR Minutes if	0.96 (0.87–1.06)	0.44	0.99 (0.87–1.09)	0.82
Total physical activity	RR Minutes if	1.29 (1.18–1.41)	<0.001	1.28 (1.17–1.40)	<0.0001
Met physical activity guidelines	OR No/yes	4.80 (2.30–10.04)	<0.001	4.10 (2.05–8.19)	<0.001

Adjustment Model 1: DO, gender, age, presence of child <16 in household, highest education achieved, work/physically active at work, Family social support for walking.

Variables tried during initial model building and found to not be required – household income, number of people, marital status, social support for walking from friends, personality measures.

Includes random effect at the household level.

The effect of dog ownership on total physical activity could not be identified from the (random) effect of household, and represents a limitation of our experimental design.

### Dog-related physical activity in children

Children’s involvement in dog walking and unadjusted (due to small sample) children’s PA comparisons by dog ownership are presented in Table 6 (n = 46). The mean child age was 10.5 years; 24 children were male and 23 children were female. Two out of ten dog-owning children (5–15 yrs) reported never walking with their dog. Again, walking for transport was less common (median 0 mins per week) than walking for recreation (median 85 mins per week), dog walking median 105 mins per week in total. Children walked their dogs a median two times during the week (median of 40 mins total), and one time at the weekend (median of 45 mins total). Three children (30%) reported running/jogging with their dog. Free-time unstructured PA (eg. playing) with the dog by children was common, with a median 205 mins per week spent in this activity (60 mins inside the house and 65 mins per week in the yard/garden). DO children reported 78 more minutes per week walking for recreation (P = 0.04), and 285 more minutes per week walking (P = 0.01) than NDO children. Free time unstructured PA (e.g playing) was also 260 mins higher in DO children (P < 0.01).

**Table 6:** Children’s (n = 46) reported physical activity (excluding activity during school time), by participants (dog –Owning (DO) and non-dog owning (NDO), in a study in West Cheshire, UK, 2015.

Outcome	n	NDO	Mean (SD)	n	DO	Mean (SD)	P Medians NDO/DO	P Means NDO/DO
		Median (IQR)			Median (IQR)			
Walk for recreation frequency/week	36	2.0 (2.8)	3.4 (6.1)	10	4 (10.5)	6.1 (6.4)	0.09	0.26
Walk for recreation mins/week	36	40.0 (105.0)	61.8 (77.2)	10	117.5 (78.8)	115.0 (97.9)	0.04	0.14
Walk for transport frequency/week	36	5.0 (7.8)	6.4 (5.9)	10	3.0 (8.3)	4.0 (4.2)	0.23	0.16
Walk for transport mins/week	36	120.0 (165.0)	143.1 (127.8)	10	52.5 (233.8)	179.0 (306.9)	0.40	0.73
Total walk frequency/week	36	6.0 (6.8)	9.9 (11.0)	10	10.5 (8.5)	10.1 (5.5)	0.32	0.93
Total walk mins/week	36	205.0 (177.5)	204.9 (140.2)	10	490.0 (488.0)	694.0 (968.0)	0.01	0.15
Freetime physical activity frequency/week (eg playing)	36	6.0 (4.8)	5.4 (3.7)	10	13.5 (13.5)	14.9 (7.1)	<0.001	**0.002**
Freetime physical activity mins/week (eg playing)	36	180.0 (230.0)	218.5 (184.0)	10	440.0 (835.0)	858 (1091)	0.004	0.10
Sports frequency/week	36	2.0 (2.0)	2.3 (1.8)	10	2.0 (3.3)	2.4 (1.9)	0.89	0.92
Sports mins/week	36	105.0 (120.0)	150.3 (183.0)	10	120.0 (207.5)	137.0 (122.8)	0.91	0.79
Total PA mins/week	36	477.5 (320.0)	565.6 (369.2)	10	680.0 (1016.0)	1035.0 (1010.0)	0.17	0.18
	n	%		n	%	OR	95% CI	P
Met children’s physical activity guidelines (excluding school activity) of 60 mins per day average	20	55.6		8	80.0	3.2	0.6–17.2	0.16

## Discussion

The odds of dog owners meeting current physical activity guidelines were four times greater than for non-dog owners. This difference (OR 4) is more marked than differences reported in other countries (OR 1.6)^12,24^. Our findings are striking when compared to a meta-analysis of typical physical activity interventions in adults which have an effect size of 0.19 (across a variety of self-report and objective measures of PA), equating to just 496 steps per day^25^. Our study also suggests that children who own dogs report greater participation in recreational walking and free time physical activity. Given that dog owners did not appear to have lower participation in other forms of physical activity compared to non-owners, our findings suggest that that adult dog owners’ increased recreational walking is contributing additional activity rather than replacing other activity. In fact, our data suggest dog owners are also more likely to participate in jogging or running without a dog than non-owners. Dog owners were less likely to report walking for transport than people without a dog, in line with previous studies^26^, but this was more than compensated for by additional recreational walking. Our novel approach to analysis elucidates that it is increased frequency of recreational walks, rather than considerably greater walk duration, explaining the principle effect of dog ownership on physical activity levels. These findings are important because guidelines recommend that activity should be frequent to break up periods of sedentary behaviour/sitting, and also undertaken in bouts of at least 10 minutes or more^3^; walking with a dog appears to be an effective strategy for facilitating this type of physical activity.

Our data confirms that people who own a dog but do not walk it (NDW) are much less physically active than both DW and NDO^27^. Only 10% of our owners reported no walking with their dog, compared to 22% in an Australian study using similar methodology^24^ and 30% in a USA study^27^, which likely contributes to our larger differences in odds of meeting physical activity guidelines. Another USA study found that only 27% of dog owners walked their dog for at least 150 minutes per week^7^, compared with 64% in the current study. We conclude that dog walking is more important to the physical activity levels of our UK community than in other countries, but a proportion of dog owners who do not walk (NDW) are pervasive. This group also have very low levels of physical activity overall. Further research is required in order to understand why and if anything can be done to facilitate their participation in dog walking. Qualitative research into barriers and motivators to dog walking suggests it may be due to owner perception of owner or dog health capabilities^11^. However, looking at the small amount of data here, NDW perhaps have a tendency to be female, under 30 yrs, working, of normal weight and self-perceived very good health.

Our study has considerable strengths over previous research. We combined self-report with validation using objective measures of physical activity, in a standardised population living in the same area, and provided novel contextual information into the types of walking and physical activity done both with and without a dog. Analysis methods were appropriate for interpretation of skewed outcomes^23^. We also collected data from multiple household members, including children, and adjusted for clustering in our analyses, demonstrating the feasibility of this approach. Thus the reliability of our findings is likely to be robust. However, the main limitation of this study is reliance on mainly self-report data (although validated measures), which could over-estimate activity levels. Findings also need to be confirmed in a larger sample and other populations. In particular, our accelerometry and child sample sizes were small. Studies of dog walking should collect both self-report and objective data, as accelerometry provides an objective measure of physical activity, whilst self-report provides more information on the context of the behaviour, i.e. walking for transport or recreation. Furthermore, as technology develops accelerometry could be combined with locational data such as GPS in order to also assess distance travelled. Finally, longitudinal studies are required to confirm causation – that dog acquisition results in increased physical activity, and that other activities are not replaced by dog walking.

In conclusion, this study provides new evidence that UK dog owners are considerably more active than people without a dog, and that dog walking is undertaken in addition to, and not instead of, other physical activities. Our study is cross-sectional in nature and cannot confirm that getting a dog *causes* people to be more active, although there is a small amount of longitudinal data which support this^28,29^. Nevertheless, the effect of dog ownership on physical activity levels in the UK appears to be greater than other countries studied. Our findings provide support for the role of pet dogs in promoting and maintaining positive health behaviours such as walking. Without dogs, it is likely that population physical activity levels would be much lower. Dog walking is also significant for wider health as physical activity undertaken outdoors and in natural environments has the greatest mental health benefits^30^, and also increases social capital through encouraging interactions in local communities^31^. Therefore our pet dogs play an important role in keeping us healthy and this should be recognised and facilitated. However, this should not be interpreted as a recommendation for people to go out and get a dog purely for their own benefit; dog welfare needs must be carefully considered. Our findings should instead be used to justify the provision of dog-supportive environments for walking and pet-friendly housing; failure of planning and policy makers to provide these may significantly damage population levels of physical activity. Findings should also be used to promote interventions to increase and maintain dog walking, as even though many owners reported significant walking with their dog, there is still potential to increase this further. It is also important to understand how to support the maintenance of the activity levels of dog walkers, in particular regarding the perceived barriers of owner and dog health and ageing^11^.

## Methods

### Participants

The study population and survey methods have been outlined previously^32^. A community of 1280 households in a semi-rural town in West Cheshire, UK, were approached up to five separate times at different days of the week and times. Interviewers (female, personable veterinary students) spoke with members of 984 households (76.9%) and for those who agreed to participate (767/77.9%), collected baseline data on household type, pets owned, and number of household members. Paper questionnaire surveys were then provided for each member of 698 households (91.0%), giving 1591 eligible participants. Participants were asked to either complete and return them by post or online. Different questionnaires were issued for adults and children (5–15 yrs). Children less than 5 years old were not surveyed due to difficulties measuring PA reliably via questionnaire in this age group. A postcard reminder was sent after 2 weeks of non-return, and a second copy of the questionnaire at 4 weeks. Survey participants were asked whether they would mind participating in further research and to provide contact details, and from this 88 people were also contacted at a later date by email/post/phone to be invited to wear an accelerometer for seven days.

### Ethical approval

The study protocol was approved by University of Liverpool Veterinary Research Ethics Committee (VREC334) and the methods were carried out in accordance with these guidelines. Households received an information flyer detailing the study a week before. Participants consented by completing and returning the questionnaires and for children ages 5–15 yrs, questionnaires were completed by the child and the parent together and posted back with the parent’s questionnaire, thus giving parental consent. The sub-sample provided informed written consent to wear the accelerometer.

### Outcomes

Physical activity items were adapted slightly from the validated RESIDE Neighbourhood Physical Activity Questionnaire (NPAQ)^33^ and Dogs And Physical Activity (DAPA) Tool^34^, to separately measure the activities with a dog of walking for recreation, walking for transport, jogging, and cycling. In summary, all participants (DO and NDO) indicated the frequency per usual week and total minutes per usual week that they engaged in walking for recreation and leisure (including for dog owners both with and without a dog), walking for transport (including for dog owners both with and without a dog), participation in other moderate intensity physical activities as defined, and other vigorous intensity physical activities as defined. The responses were used to calculate frequency and minutes dog-related physical activity, total walking, total recreational walking, total transport walking, MVPA, and total PA per week, as well as percentage contributions to total PA of the various components of walking.

Children’s PA questions were completed by the child with the parent and used a modified version (to include activities with and without dogs) of the questions used for children in the Child and Adolescent Physical Activity and Nutrition Survey (CAPANS)^35^. In brief, questions asked about frequency and total minutes spent in each activity type in a usual week (mon-fri), and weekend (sat-sun), undertaking: (a) walking without your dog for recreation, health or fitness; (b) walking without your dog for transport; (c) playing sport or structured physical activity; (d) free-time unstructured activity without your dog; (e) walking with your dog for recreation health or fitness; (f) Walking with a dog as a means of transport; (g) jogging or running with a dog; (h) free time activity with your dog in the backyard/garden; (i) free time activity with your dog inside the house; (j) other activity with your dog.

A subset of 31 adults and 3 children also wore Actigraph GTX3 accelerometers for 7 days within six months of completing surveys. The monitor was worn on the right hip during waking hours and recorded at 1 second epochs. Only adult data was further processed. Diaries were used to validate periods of non-wear. Valid data of at least 3 full days wear (1 weekend, 2 weekday, at least 500 mins per day) was available for n = 28 adults and activity intensities were classified by converting the data to 60 second epochs and then using validated cut points for adults^36^. From this an estimation of total time spent in PA for a 7 day week (minutes) was calculated. These data were used to classify whether or not the participant met guidelines of 150 minutes MVPA per week (yes/no) and this was then compared with the self-report survey data in order to highlight whether there was considerable over-reporting

### Variables

Socio-demographic data collected included (see Table 1): house type; number of people in the household; children <16 present in household; current age of participant; gender; highest education level; occupation; household income; dog ownership; marital status; work status; and PA at work. Other questions included: self-rated general health; height and weight (used to calculate BMI and categorise as normal, overweight or obese); Ten Item Personality Inventory (TIPI)^37^; social support from family and friends for walking^33,38^.

### Statistical analysis

Simple descriptive analysis (from both self-report and accelerometry data) was conducted using Kruskal-Wallis tests for continuous outcomes, because the PA outcome data was highly skewed. We have also presented means and t-test findings for comparison because these are often presented in similar PA studies, despite not being appropriate due to non-normality of the data. Chi-squared tests were used to compare proportions between groups. Parametric linear regression methods are also not strictly appropriate for analysis of PA data, despite often being undertaken^23^. Due to zero-inflation in categories where a respondent often reported zero activity, simple transformation methods also did not normalise the data. To address this problem, two analyses were conducted; (1) binary logistic regression was used to fit models with binary outcomes (ie whether or not that activity type was reported); and (2) where an activity was undertaken, linear regression on that subset of participants was used to fit models with the outcome measured as log10-transformed minutes of PA per week. This transformation was chosen to satisfy Normality of the model residuals, with coefficients interpreted as relative risks (RR) calculated for DO against NDO. This allowed comparison of the likelihood of an activity being undertaken at all (OR), and the duration per week the activity occurred if it was undertaken (RR interpreted as % difference in minutes). Due to non-independence of data, participants belonging to the same household were adjusted for using a random effect at the household level.

Univariable analyses were conducted to explore potential confounding of the relationships between measures of PA and dog ownership, to inform the model building process. All models included the independent variable of interest – dog ownership. Three levels of models were developed for each outcome (See Table 5); Model 1 – sociodemographic factors and social support factors identified through the univariable analysis and retained through backwards selection (gender was non-significant at P < 0.05 but deemed important to retain); Model 2 – addition of weight status; Model 3 – addition of self-reported general health. Models including weight status and self-reported perceived general health were tested due to the reasoning that being overweight or in poor health could be both a cause and outcome of low PA levels. Modelling was conducted in R v3.3.0 and the *nlme* R library.

## Data Availability

Please contact the corresponding author for requests for access to anonymised data.
